# Alternatively Splicing Interactomes Identify Novel Isoform-Specific Partners for NSD2

**DOI:** 10.3389/fcell.2021.612019

**Published:** 2021-02-25

**Authors:** Weidi Wang, Yucan Chen, Jingjing Zhao, Liang Chen, Weichen Song, Li Li, Guan Ning Lin

**Affiliations:** ^1^Shanghai Mental Health Center, Shanghai Jiao Tong University School of Medicine, School of Biomedical Engineering, Shanghai Jiao Tong University, Shanghai, China; ^2^Shanghai Key Laboratory of Psychotic Disorders, Shanghai, China

**Keywords:** NSD2, alternatively splicing, protein–protein interaction, isoform, RPL10

## Abstract

*Nuclear receptor SET domain protein* (*NSD2*) plays a fundamental role in the pathogenesis of Wolf–Hirschhorn Syndrome (WHS) and is overexpressed in multiple human myelomas, but its protein–protein interaction (PPI) patterns, particularly at the isoform/exon levels, are poorly understood. We explored the subcellular localizations of four representative *NSD2* transcripts with immunofluorescence microscopy. Next, we used label-free quantification to perform immunoprecipitation mass spectrometry (IP-MS) analyses of the transcripts. Using the interaction partners for each transcript detected in the IP-MS results, we identified 890 isoform-specific PPI partners (83% are novel). These PPI networks were further divided into four categories of the exon-specific interactome. In these exon-specific PPI partners, two genes, RPL10 and HSPA8, were successfully confirmed by co-immunoprecipitation and Western blotting. RPL10 primarily interacted with Isoforms 1, 3, and 5, and HSPA8 interacted with all four isoforms, respectively. Using our extended NSD2 protein interactions, we constructed an isoform-level PPI landscape for NSD2 to serve as reference interactome data for NSD2 spliceosome-level studies. Furthermore, the RNA splicing processes supported by these isoform partners shed light on the diverse roles NSD2 plays in WHS and myeloma development. We also validated the interactions using Western blotting, RPL10, and the three NSD2 (Isoform 1, 3, and 5). Our results expand gene-level NSD2 PPI networks and provide a basis for the treatment of NSD2-related developmental diseases.

## Introduction

*The nuclear receptor SET domain containing protein 2* (*NSD2*), also known as *MMSET* or *WHSC1*, is a member of the NSD protein family (Allali-Hassani et al., 2014), which mainly catalyzes histone H3 lysine (Huang et al., 2013; Poulin et al., 2016). *NSD2* is a critical gene in the pathology of Wolf–Hirschhorn Syndrome (WHS; Jiang et al., 2019; McDevitt et al., 2019), a severe neurodevelopmental disorder characterized by distinctive developmental delays (DDs), intellectual disabilities (IDs), and seizures, which occur in more than 50% of WHS infants (Deardorff and Zackai, 2007). The disease results from distal deletions on the short arm of chromosome 4 (chromosome 4p16.3) (Descartes et al., 2017), which occurs in 1 in 50,000 births (Deardorff and Zackai, 2007). *NSD2* carries rare mutations in patients with neuropsychiatric disorders, including autism spectrum disorder (ASD), DDs, IDs, and schizophrenia (SCZ; Boczek et al., 2018; Park et al., 2018; Barrie et al., 2019; Lin et al., 2019). Early studies have reported various deleterious *NSD2* variants in neuropsychiatric patients, suggesting that the haploinsufficiency of *NSD2* might be partially responsible for DDs (Katoh, 2016; Kim et al., 2017; Derar et al., 2019). However, over the past decade, most functions of *NSD2* have been identified in carcinogeneses, such as renal cell carcinoma (Han et al., 2019), colorectal cancer (Chen et al., 2019), osteosarcoma (He et al., 2019), and multiple myeloma (Kuo et al., 2011). The biological divergence and potential mechanistic differences in *NSD2* associated with neurodevelopmental disorders and cancers remain undetermined.

Compelling data have emerged to support the concept that alternatively spliced isoforms are linked to a range of functional characteristics of certain genes (Kelemen et al., 2013; Marshall et al., 2013; Chaudhary et al., 2019) and contribute to functional complexity in diseases (Yang et al., 2016; Narasimhan et al., 2018). The alternative splicing of pre-mRNAs is widespread in humans and most eukaryotes (Modrek and Lee, 2003; Park et al., 2018), and it happens in ∼95% of genes containing different numbers of exons (Pan et al., 2008; Wang et al., 2008). There are striking functionally diverse gene functions in human brains in particular due to alternative splicing (Graveley, 2001). Although alternative splicing is generally responsible for the diversity of gene products expressed from the genome, the complexity of alternative splicing at the proteome level remains to be characterized (Blakeley et al., 2010). In addition, large-scale proteomics experiments are usually only focused on a single gene-level protein approach to simplify the number of proteins for use in further analyses. Moreover, most large-scale experiments have relied on antibodies recognizing a region common to different isoforms or have chosen the most characterized protein isoform to include to identify protein interactions or expression patterns. However, studies on NSD2 isoforms have been limited (Huang et al., 2013). *De novo* variants (DNVs) play a vital role in understanding the genetics of psychiatric disorders (Veltman and Brunner, 2012). There have been 52 DNVs found in psychiatric patients, including nine DNVs that affect protein-coding regions (Lin et al., 2019). Among these exonic DNVs, two are found in ASD (C Yuen et al., 2017; Stessman et al., 2017), five in DDs (Deciphering Developmental Disorders Study, 2017) or IDs (Lelieveld et al., 2016; Heyne et al., 2018), and only one in SCZ (Howrigan et al., 2018) and congenital heart disease (Homsy et al., 2015). These DNVs affect different exons among NSD2 isoforms, which contribute to phenotypic differences.

In addition, integrating protein–protein interactions (PPIs) to study the potential functional impacts of risk genes on the associated disorders at the level of the biological system is a common practice in the study of disease biology (Corominas et al., 2014; Zhang et al., 2018; Oughtred et al., 2019). For example, ASD-associated physical interaction networks formed by protein interactome, which focus on cancer-related genes such as β-catenin (O’Roak et al., 2012), p53 signaling (O’Roak et al., 2012), Wnt–β-catenin (Krishnan et al., 2016), and MAPK (Krishnan et al., 2016), have provided important insights into the interpretation of diseases. In addition, protein interactomes can also be useful for investigating key pathways, such as abnormal synaptic phenotypes (Noh et al., 2013) and post-synaptic density (Krishnan et al., 2016) in ASD focusing on targeted functional gene sets. Using 343 WHS-associated genes, including NSD2 itself, Corrêa et al. (2018) have constructed a PPI network with GeneMANIA (Franz et al., 2018), which was able to identify a gene set with a role in NAD+ nucleosidase activity. However, PPIs at the gene level may not completely reflect the complex system underlying disease etiology, particularly because several studies have shown that different isoforms of the same gene can differ in both biological function and subcellular component (Messaoudi et al., 2007; Ilouz et al., 2017). In addition, it has been shown that alternatively spliced isoforms of the same gene can have different sets of interaction partners (Corominas et al., 2014), and the interactome analyses of the isoforms of interest would facilitate the identification of their functional biological roles (Yang et al., 2016).

We also found that the interactors for NSD2 shed light on the function of histone methyltransferase activity (Huang et al., 2019), and they supply resources in constructing the interactome of NSD2 (Gordon et al., 2020; Luck et al., 2020). However, these interactome analyses of NSD2 have mostly remained at the gene level, limiting downstream functional analyses and leaving the PPI patterns at the isoform/exon levels poorly understood. Indeed, the main genomic databases, e.g., RefSeq (O’Leary et al., 2016) and Ensembl (Zerbino et al., 2018), apply different identifiers for *NSD2* transcripts, and previous reports have merely specified which transcript or protein isoforms are under consideration (Allali-Hassani et al., 2014; Haladyna et al., 2015; Aytes et al., 2018; Ouda et al., 2018). A few studies have focused on *NSD2* transcript variant 1 (accession number: NM_133330.2) (Jiang et al., 2019), but the remaining isoforms of *NSD2* have been poorly explored. Therefore, isoform-specific interactome analyses of NSD2 are urgently needed.

To fill this gap and yield further insight into the divergent etiology of *NSD2* isoforms, we experimentally screened selected exon-specific isoforms of *NSD2* and their protein-level interactions, then systematically incorporated the interactions into a study of the biological network to investigate potential functional interaction networks for different *NSD2* isoforms (Supplementary Figure 1). We selected four representative transcripts, namely, Isoforms 1, 3, 5, and 7, and then we used label-free quantification to perform immunoprecipitation mass spectrometry (IP-MS) analyses. Using isoform-specific partners, we analyzed the diversity between the differential networks of NSD2 cleavage isomers and explored the underlying molecular functional pathways. We identified 365 proteins as novel interactors with NSD2 isoforms. These novel partners were significantly enriched in proteins that are functionally characterized as RNA splicing, in addition to literature-reported NSD2 interactors, whose functions relate to the chromatin remodeling. One of the novel partners, the ribosomal protein gene RPL10, a genetic factor to cognitive function (Klauck et al., 2006), was specially partnered by Isoform 1, 3, and 5. We further validated the interaction between RPL10 and NSD2 isoforms experimentally using Western blotting, supporting the credibility of our data and extending the knowledge of the protein partners of NSD2.

## Results

### Cloning, Expression, and Localization of *NSD2* Isoforms

**FIGURE 1:**
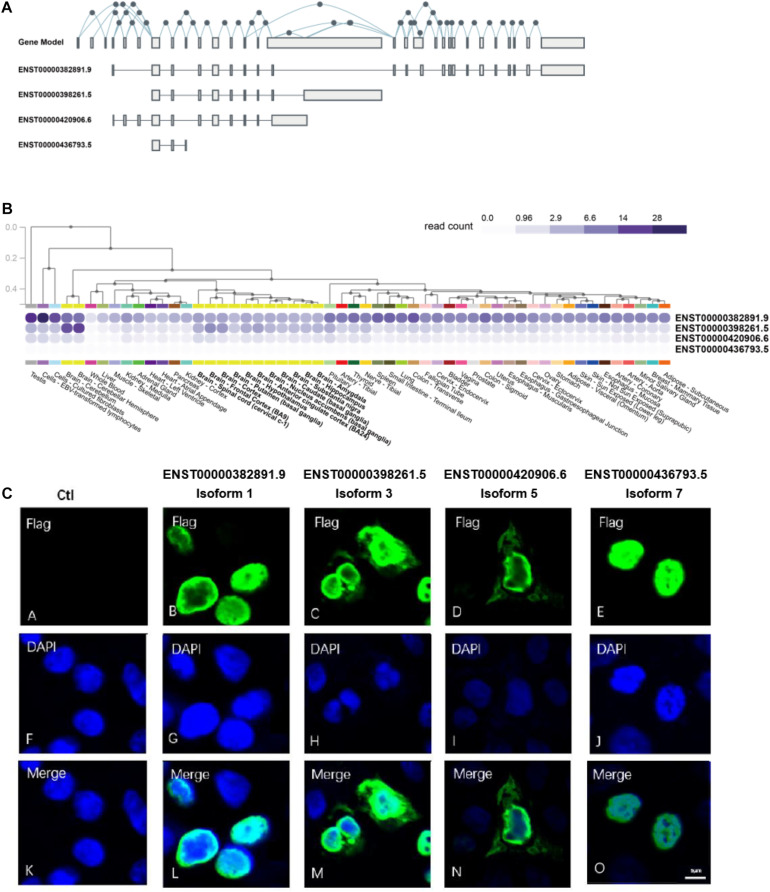
*NSD2* transcripts and protein isoforms have different expression patterns. **(A)** The gene model and exon distributions in transcripts of *NSD2*. **(B)** Four transcripts of *NSD2* were expressed in human tissues by using the GTEx database. **(C)** The subcellular locations of four protein isoforms of *NSD2*.

Alternative splicing of exons resulted in 27 *NSD2* transcripts, of which 14 can be translated into proteins.^1^ We selected four of them because of their mutually excluded exons and domains (Figure 1A): Isoform 1 (ENST00000382891.9) contains exons 4, 7, 8, 10–15, and 17–29; Isoform 3 (ENST00000398261.5) contains exons 7, 8, 10–15, and 16; Isoform 5 (ENST00000420906.6) contains exons 4–8 and 10–15; Isoform 7 (ENST00000436793.5) contains exons 7–9 (Supplementary Figure 2). We named our NSD2 isoforms using the same IDs from the UniProt (UniProt Consortium, 2019). It is worth noting that all isoforms except for Isoform 1 have unique exons. For example, Isoform 3 uniquely contains exon 16, Isoform 5 uniquely contains exons 4–6, and Isoform 7 uniquely contains exon 9.

It has been reported that tissue-specific expression patterns happen in a substantial proportion of isoforms generated from the same gene due to alternative splicing events (Barbosa-Morais et al., 2012; Merkin et al., 2012; Yang et al., 2016). To establish the differences among the four isoforms of *NSD2* investigated in this study, we first looked at the transcript-level expressions for four isoforms using GTEx (Ardlie et al., 2015). We observed that the transcripts have specific expression patterns across the tissues of the testis, bone marrow, lymph node, and brain (Figure 1B). The highest level of expression was detected in Isoform 1, followed by Isoforms 3 and 5, while Isoform 7 was expressed at a very low level. Based on the transcript-level quantifications of bulk RNA-Seq, the expression of Isoform 7 in tissues was not detected. We also used quantitative real-time polymerase chain reaction (qRT-PCR) to confirm the expression levels of Isoform 7 in the HEK293T cell line, even at a low level (Supplementary Figure 3). Collectively, the variation in expression patterns of the *NSD2* isoforms strongly suggests their functional divergence.

Subcellular localization could indicate the extent to which enzymatic activities can be regulated by the products of isoforms (Kobayashi et al., 2007; Nair and Rost, 2009; Du et al., 2018). To explore the potential function of each protein isoform, we performed the subcellular location detection and investigated localization characteristics of the four NSD2 isoforms via immunofluorescence (IF) microscopy (Figure 1C). Each isoform vector was first transfected into HEK293T cells, and the expression of each subtype was induced. The cells were then fixed before being subjected to IF labeling using FLAG antibody, and the nuclei were observed with DNA staining using DAPI. As expected, the imaging of the isoforms showed convergent and divergent subcellular distributions (Figure 1C). These data showed whether isoforms expressed in the nucleus, with the nuclear accumulation of Isoforms 1 and 7 and cytoplasmic and nuclear accumulations of Isoforms 3 and 5.

### NSD2 Isoform Interactomes in HEK293T Cells

**FIGURE 2:**
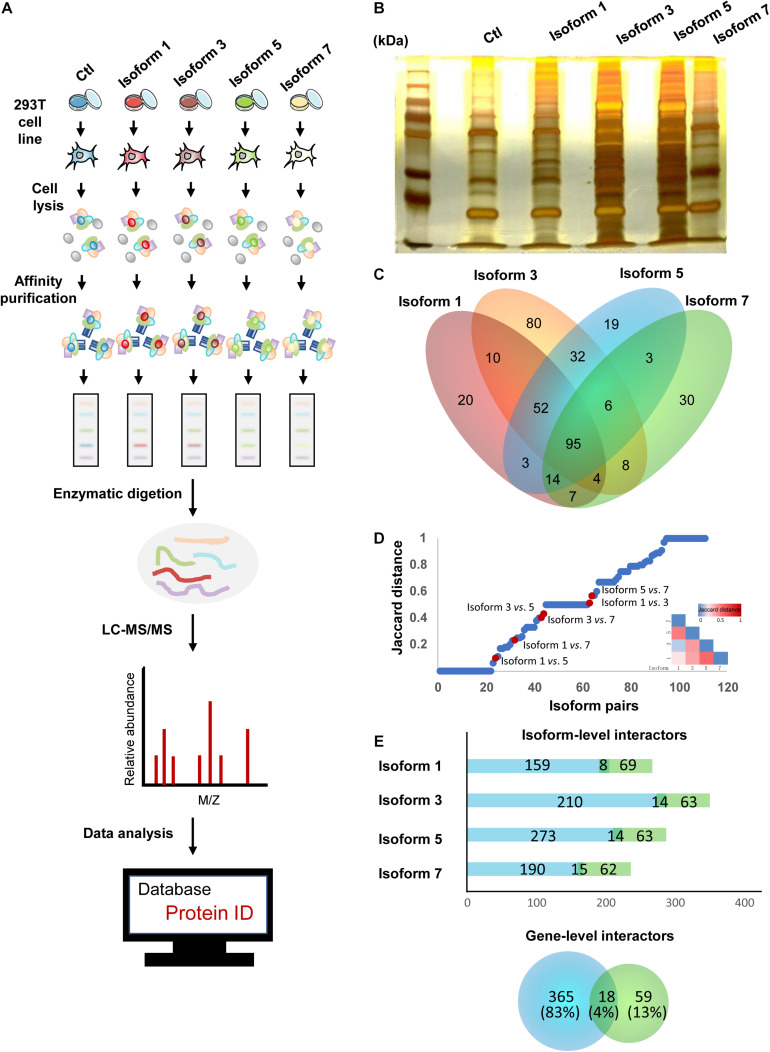
NSD2 isoform-level interactors exhibit importance in the interactome network. **(A)** The pipeline of IP-MS to identify interactions. **(B)** Silver stainings of NSD2 isoforms. **(C)** The diagram of overlapped partners between isoforms. **(D)** Distribution of paired NSD2 isoforms in the interaction profile differences between all possible pairs of alternative isoforms from Yang et al. (2016). The Jaccard distance of 0 means that both isoforms share all interaction partners, whereas a distance of 1 means the isoforms have no shared partners. The above heatmap shows the value of Jaccard distance in paired isoforms of NSD2. **(E)** The histogram represents the number of the isoform-level interactors according to the isoform type, reference (light green), or non-reference (light blue). The Venn diagram below represents the number of the gene-level PPIs (a total of 442), reference (light green), or non-reference (light blue). Some genes have PPIs shared by the reference and the non-reference isoforms.

Previous studies of alternative splicing isoforms and their PPIs have shown that different isoforms of the same proteins can have variable biological functions, ranging from similarities in binding partners to completely different sets of partners (Corominas et al., 2014; Yang et al., 2016; Lin et al., 2017). In addition, due to the differences in their tissue expression and subcellular localization, we hypothesized that NSD2 isoforms could also vary in their protein binding targets. To investigate isoform-specific interactors, *in vitro* affinity-capture assays, coupled with label-free quantification of interacting proteins, were performed in HEK293T cells using isoform-Flag recombinant proteins as bait and Flag alone as a control to subtract non-specific interactions (Figure 2A). We first constructed four expression vectors for NSD2 Isoforms 1, 3, 5, and 7, and a blank vector served as control. Then we transfected different isoform vectors into 293T cell lines and tested the isoform vectors’ stably expressed condition to ensure that there was no degradation (Figure 2B). To identify the interacting partners of NSD2 isoforms, we used label-free quantification to perform MS analyses of the isoform and control sample and obtained IP-MS data for the five samples. These experiments were done in triplicate. To validate the overall quality of the MS results.

Isoform-specific interactors were identified by comparing the MS results for NSD2 isoforms and control, and we obtained 383 protein partners interacting with the four isoforms encoded by NSD2. First, we filtered the detected partners with the cutoff value of the peptide frequency that occurred more than once in a single test and appeared twice or more in the three biological repeat tests. We found that Isoform 1 had 205 interacting partners, Isoform 3 had 287, Isoform 5 had 224 interacting, and Isoform 7 had 167. There were overlapping protein partners between these isoforms established by comparing the different isoforms (Figure 2C). Finally, we identified 20 interacting partners specific to Isoform 1, 80 to Isoform 3, 19 to Isoform 5, and 30 to Isoform 7 by comparing the unique protein partners.

To investigate the extent to which the four isoforms mediate interactions with different partners, we evaluated the dissimilarities in their interaction profiles by calculating the Jaccard distance of every pairing of four isoforms (Figure 2D). We restricted our analyses by comparing our paired NSD2 isoforms with the validated interaction of the 105 isoforms reported by Yang et al. (2016). Globally, we found that most isoform-specific protein partners have not been reported, except for 18 overlapping interactions between the gene level and the isoform level. By comparison with the NSD2 interactors obtained from BioGrid (Oughtred et al., 2019), we found that a substantial proportion of interacting partners was exclusively identified in our isoform-level data. Only 4% of the partners were repeated at both the gene and isoform levels. Another 13% were found in gene-level PPIs, and the remaining 83% were only identified at the isoform level (Figure 2E). The targeted isoform partners exhibited a shrinking percentage of PPIs (67–78%), as the equal proportions of novel interactors showed by other reports (Corominas et al., 2014; Yang et al., 2016), emphasizing the importance of isoform-level exploration for protein interaction networks. As Huang et al. identified high confidence NSD2 interacting partners in MM cells (Huang et al., 2019), we compared our interactors of NSD2 isoforms with their NSD2 partner proteins and found the resemble trend that NSD2 partners in isoform level contribute more in the interaction network.

### NSD2 Isoform-Specific Partners That Indicate Distinct Disease Mechanism

**FIGURE 3:**
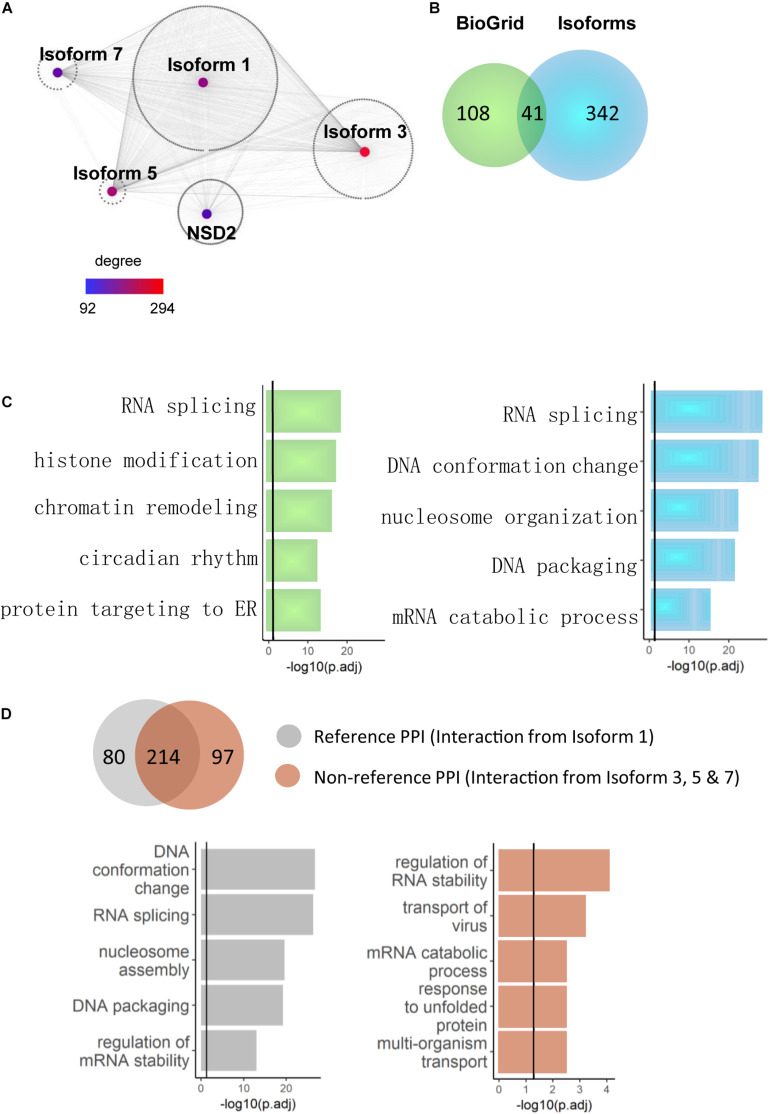
NSD2 isoform-specific function enrichments. **(A)** The network of the gene and isoform level partners of NSD2. The heatmap shows the degree of each node (Isoform 1, 3, 5, 7, and NSD2). **(B)** The Venn diagram shows the overlapped interactors between NSD2 isoforms and reference interactors of NSD2 from BioGrid. **(C)** The enrichment results of BioGrid and our isoform interactors. The histogram in light green represents genes from BioGrid, and the light blue represents genes from our NSD2 isoform interactions. **(D)** The Venn diagram shows the overlapped PPIs between Isoform 1 and the other three isoforms. The histogram represents the enrichment categories of both interactors.

Because novel NSD2 interactors were primarily found at the isoform level, we explored whether the interactome network construction of these interactors illustrated different functional pathways. Because the interacting partners of a single node (gene/isoform) in a network could have notably different properties than those of proteins that interact with separate nodes (Corominas et al., 2014), we reasoned that combining the direct binary partners of NSD2 isoforms in gene/isoform subnetworks may reveal functional differences between them. Based on the literature-reported partners curated from BioGrid (Oughtred et al., 2019), we first extended our analyses by merging both novel and literature-reported partners into the pool to construct a gene-isoform network for NSD2 (Figure 3A). This extended network included all 991 interactions and combined with ∼90% of isoform-specific partners globally (Figure 3B). In the resulting network, Isoform 5 reached the degree of 294, the highest found. The gene NSD2, which is only equipped with literature-reported PPIs, had only 92, the lowest degree. This expanded protein interaction capability of NSD2 suggests a functional divergence among the four isoforms.

We examined whether the isoforms’ extended protein networks were significantly enriched in a range of functional categories involved with disorders to investigate this functional divergence. For the literature-reported NSD2, the significant enrichment terms include RNA splicing, chromatin remodeling and histone modification (Figure 3C), in which NSD2 has been documented to play an important role (Mirabella et al., 2014). For our isoform specific interaction partners, RNA splicing and DNA conformation featured the most significant enrichment and showed more importance than other functional terms. This accumulating evidence suggests that the isoform-specific interactions of NSD2 play vital roles in the subnetwork.

To confirm the relationship between RNA splicing and NSD2 isoforms, we divided our interactome into Reference PPIs, which are interactions of Isoform 1 that has been commonly used to represent NSD2 in gene-level studies, and Non-reference PPIs, which are interactions from all other isoforms. The varied results of enrichment suggest caustic usage of NSD2 isoforms (Figure 3D). Isoform 1 interactions were significantly enriched in DNA conformation and RNA splicing. By contrast, the other three isoform interactions were enriched in regulating RNA stability and transport of virus and differed from the Reference PPI. Indeed, the isoform PPIs of the NSD2 indicate a potential distinguishing underlying mechanism, particularly for these exon-specific matched DNVs from patients with different psychiatric disorders.

### Isoforms Specific to NSD2 Exon Junctions Associated With Distinct Pathways

**FIGURE 4:**
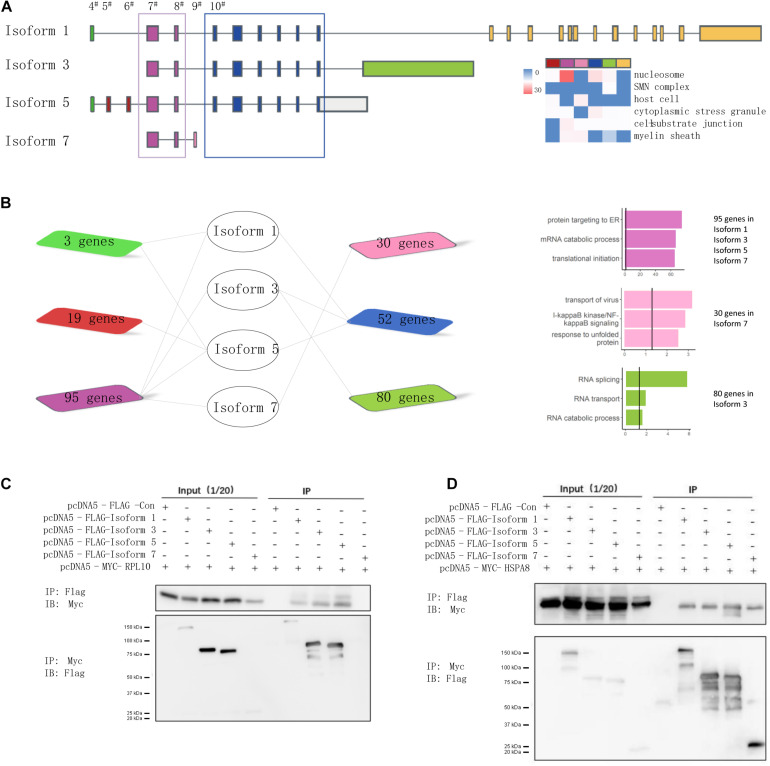
NSD2 exon junction specific clusters show different function. **(A)** The junctional patterns of exons into clusters. **(B)** The diagram shows the shared genes between exon specific isoforms, and the histogram in the same color represents enrichment results corresponding to the diagram genes. **(C)** Western blots of all four NSD2 isoforms and RPL10. **(D)** Western blots of all four NSD2 isoforms and HSPA8.

Because splicing could mediate the disruption of interactions through its inclusion or exclusion of domains, the targeted domains can be predicted to interact with interacting partner proteins that contain a certain region (Mosca et al., 2014; Yang et al., 2016). Following the hypothesis that isoforms holding the same exons share a regulation or function (Richard et al., 2010), we prioritized them based on the exon composition of the included isoforms to investigate the role more deeply of alternative exons in isoform-level interactions of NSD2. We proposed that the isoforms that constituted the same exons could have the same protein domains and/or similar biological functions. We considered the difference between NSD2 isoforms that include or exclude a target exon. For this purpose, we performed analyses to categorize isoforms from the census exon into clusters (Figure 4A). This procedure divided the interacting partners into seven groups that shared different numbers of genes (Figure 4B). We found that the cluster for exon 7–8 was mainly concentrated in the protein targets to ER, mRNA catabolic process, and translational initiation; the cluster for exon 4 was mainly related to RNA splicing, and RNA transport; and the binding proteins in the group for exon 9 were enriched in transport of virus, NF-kB signaling pathway, and response to unfolded proteins.

To examine the consequences of the exon-specific partners of isoforms, we selected two representative genes, RPL10 and HSPA8, for their presentation in the subnetworks constructed by Isoforms 1, 3, and 5 and in all four isoforms separately (Figure 4B). Because the domain PWWP 1 was included in Isoforms 1, 3, and 5 but not Isoform 7 (Kim et al., 2007), the binary interaction with RPL10 suggested that isoform-specific partner differences could be explained by the alternative splicing of protein domains. RPL10, located on the Xq28 chromosome, has an essential function in ribosome assembly and protein translation, and it is associated with neurodevelopmental disorders (Gong et al., 2009; Zanni et al., 2015). We then explored the protein interactions between different isoforms of NSD2 and RPL10 by co-immunoprecipitation (Figure 4C). The result showed that RPL10 could bind to Isoform 1, 3, and 5, but there was no detectable interaction between RPL10 and Isoform 7. Moreover, the interactions between HSPA8 and the four isoforms were also confirmed by co-immunoprecipitation, which supported our findings in the IP-MS data that all four isoforms interacted with HSPA8 (Figure 4D).

## Discussion

We identified functional diversity in NSD2 isoforms and implicated several novel protein interactors associated with NSD2. We also verified novel protein partners via co-immunoprecipitation. Similar to the results of other studies of expanding protein interaction networks by isoform-specific level (Corsetti and Azpiazu, 2013; Corominas et al., 2014; Tseng et al., 2015), our data indicate a significant extension of interactors to NSD2, showing different enrichment categories at the isoform level.

*Nuclear receptor SET domain protein* encodes a protein that contains four domains: a PWWP domain, an HMG box, a SET domain, and a PHD-type zinc finger (Stec et al., 1998). The *NSD2* gene has 29 exons, which are combined in different ways to construct 27 transcripts. The alternative splicing of NSD2 results in multiple transcripts encoding for different protein isoforms. Since some transcripts are nonsense-mediated mRNA decay candidates, they are not represented as reference sequences.

It has been demonstrated that *NSD2* is strongly associated with tumorigenesis by promoting histone methylation, which is crucial for transcriptional regulation and chromatin remodeling (Kim et al., 2008; Han et al., 2019; Tanaka et al., 2020). *NSD2* alternative splice isoforms in cancers, in particular, have received substantial attention as of late (Huang et al., 2013; Clayton et al., 2020). The deletion of NSD2 can cause WHS (Morishita and Di Luccio, 2011; García-Carpizo et al., 2016), which has clinical characteristics that overlap with ASD. The genetic etiology of ASD is heterogeneous, attributed to hundreds of genes, of which only a small percentage has sufficient evidence to support being considered as a cause (Sanders et al., 2012; Sjaarda et al., 2020). In this study, we have systematically incorporated the interactions of the isoforms of the selected candidate gene *NSD2* into a biological network study. By inspecting the convergence and divergence of the differential network of NSD2 isoforms, we explored functional molecular pathways related to NSD2, which can serve as new therapeutic targets for ASD.

To interpret DNVs at the NSD2 isoform level, we selected four representative transcripts because their exon junctions are responsible for alternative splicing. We also curated likely damaging DNVs from PsyMuKB and found their specific mappings in the above three transcripts, except Isoform 7, which does not include exons 10–15, where there were four nonsense variants explored in diseases (Boczek et al., 2018; Park et al., 2018; Barrie et al., 2019; Lin et al., 2019). It is worth mentioning that the SET domain-containing protein 2 gene, which has the same domain as NSD2, has also shown a *de novo* gene-damaging mutation through whole-exome sequencing (Lumish et al., 2015). We also found that NSD2 is expressed across different tissues, which indicates that its dysregulation may lead to disease. In conclusion, our data emphasize that isoform specificity plays a critical role in the various biological processes. The state of differentiation for each NSD2 isoform resides in the exon junction methods. As the mutations that affect various isoforms have different exons, they have different impacts on the isoforms of the same gene (Zhang et al., 2014). The four varied N-terminus representative isoforms (Isoforms 1, 3, 5, and 7) examined here enabled us to explore exon junction clusters, a prerequisite for the functional enrichment of interactors.

NSD2 may play a role in regulating ribosome assembly and protein translation by binding its partner RPL10. Because RPL10 disrupts neurodevelopment, and a rare mutation of it has been found in ASD (Anderson-Schmidt et al., 2013; Angelova et al., 2018; Keil et al., 2018; Takumi and Tamada, 2018), its connection with NSD2 also implies that both genes may contribute to ASD. Moreover, the interaction between NSD2 and RPL10 has been previously identified by Huang et al. (2019). We have also validated the interaction between them with Western blotting. In this study, we did not carry out a detailed examination of NSD2 regulation of substrates’ stability, such as HSPA8 and RPL10, which could be performed in subsequent work where the specific disease pathway and upstream and downstream analyses of NSD2 in ASD can be clarified to establish the underlying mechanisms.

One limitation of the study is the absence of verified interactions between NSD2 isoforms and partner proteins in physiological conditions. As the PPIs confirmed under physiological conditions will enhance the understanding of their functions *in vivo*, the interactome of our isoforms could provide a straightforward functional annotation for NSD2. Unfortunately, as the commercial antibodies for these isoforms are not available, we therefore have chosen to valid their interactions of exogenous isoforms and their partners in this study. Even though these interactions of exogenous proteins might not simulate the biological systems directly, our present results could still provide some preliminary insights into the protein interaction exploration.

To the best of our knowledge, this study was the first time that the relationship between different gene-level and isoform-level interactions of NSD2 were elucidated and where the difference between functional enrichments was established. Our data indicate that interactor partners are significantly expanding at the isoform level, and different metabolic pathways are found beneath DNV-induced disorders. The use of conformational extension to induce interactors at the isoform-level makes this elusive network PPI generally available and has the potential to shed light on the biological pathways underlying a range of developmental disorders.

## Materials and Methods

### Plasmids and Reagents

NSD2 full-length plasmids were gifts from Lili lab. Full-length and other isoforms of NSD2 were PCR amplified and cloned into pcDNA5-Flag to generate Flag-tagged fusion proteins. RPL10 and HSPA8 were cloned into pcDNA5-Myc to generate Myc-tagged fusion proteins. These constructs were cloned into pcDNA5 using *Bam*HI and *Xho*I restriction sites. N-terminal Polη truncations were generated with 5’ and 3’ primers containing *Bam*HI and *Xho*I restriction sites, respectively, and cloned into pcDNA5. Primers were used:

**Table:** 

1-NSD2-F-*Bam*HI	CGCGGGATCCATGGAATTTAGCATC
1-NSD2-R-*Xho*I	CGCGCTCGAGCTATTTGCCCTCTGT
3-NSD2-F-*Bam*HI	GGATCCATGGAATTTAGCATCAAGCA GAGTCCCCTTTCTGTTCAGAGTGTTG TAAAGTGCATAAAGATGAAGCAGGC
3-NSD2-R-*Xho*I	CTCGAGCTAAGTGCAGTACAGAGCAG CTGGGTTCAAATCCAACTTGACTGGT GTGGGCTCCCACAAAAGC TCATTCTCAGTTAAGGA
5-NSD2-F-*Bam*HI	CGCGGGATCCATGGAATTTAGCATC
5-NSD2-R-*Xho*I	CGCGCTCGAGTTATTTTACCTCATT CTCAGT
7-NSD2-F-*Bam*HI	GGATCCATGGAATTTAGCATCAAGCA GAGTCCCCTTTCTGTTCAGAGTGTTG TAAAGTGCATAAAGATGAAGCAGGC
7-NSD2-R-*Xho*I	CTCGAGTTAATCTTTCAGTACAATTT GACTTGTTTTTAAGTGTTCAAACTTC TTTGATTTGAAAATACCTTTAAGTTT GGTATAGCTG

Anti-Flag M2 agarose affinity gel was purchased from Sigma (#A2220). Antibody against Myc was from Cell Signaling Technology (#2276). Antibody against Flag was from Cell Signaling Technology (#14793).

### Cell Culture and Reagents

293T cells were obtained from Lili lab. All cells were cultured in DMEM medium supplemented with 10% fetal bovine serum (FBS) at 37°C in the presence of 5% CO_2_ if not specified. For transient transfection experiments, cells were transfected with indicated constructs using jetPRIME (Polyplus-transfection) following the manufacturer’s protocol. Forty-eight hours later, transfected cells were collected for further experiments.

### Quantitative Real-Time Polymerase Chain Reaction

RNAs were extracted with Trizol reagent (Thermo Fisher Scientific) according to the manufacturer’s instructions. The cDNA was obtained using PrimeScript RT reagent Kit with gDNA Eraser (Perfect Real Time) (Takara). Primers were designed using Primer3 version 4.0.0. The qRT-PCR assay was performed using a 20-μl reaction system with SYBR Green Master reagents (Roche) and the designed primer mixtures in ABI 7900 HT Real-time PCR system (Applied Biosystems). The reaction system contained 10 ul SYBR Green Master (ROX), 0.2 μl of each primer (10 μM), 2 μl template (about 25 ng/μl cDNA) and ddH_2_O. Initial denaturation was at 95°C for 5 min followed by 40 cycles of 95°C denaturation for 10 s, 55°C annealing for 20 s, and 72°C extension for 20 s. GAPDH was used as an internal control. Relative quantification (RQ) was derived from the cycle threshold (Ct) using the equation RQ = 2^–Δ^ ^Δ^ ^*Ct*^. The forward primer for Isoform 5 is 5′-ACCCATCAGAGTGTTCTA-3′, and reverse is 5′-GTGCCTGCTTCATCTTTA-3′. The forward primer for Isoform 7 is 5′-GACCACCTGTTGAAATAC-3′, and reverse is 5′-TCTTTGATTTGAAAATACCTTTA-3′.

### Immunofluorescence

Briefly, cells were seeded on cover glasses and irradiated with ultraviolet C (UVC). The cells were permeabilized with 0.5% Triton X-100 for 5–30 min before being fixed in 4% paraformaldehyde. The samples were then blocked with 1X PBS/5% normal serum/0.3% Triton™ X-100 for 60 min. The cells were next incubated with indicated antibodies overnight at 4°C, followed by incubation with Alexa Fluor 568 goat anti-mouse (Invitrogen, Molecular Probes) for 60 min. The cells were later counterstained with DAPI, and images were acquired with a Leica DM5000 (Leica) equipped with HCX PL S-APO 63 × 1.3 oil CS immersion objective (Leica) and processed with Adobe Photoshop 7.0.

### Co-immunoprecipitation and Western Blotting

HEK293T cells were transfected with Flag-NSD2 and Myc-RPL10 or Myc-HSPA8. Forty-eight hours later, the cells were harvested and lysed with Cell lysis buffer for Western and IP (20 mM Tris (pH 7.5), 150 mM NaCl, 1% Triton X-100, and sodium pyrophosphate, β-glycerophosphate, EDTA, Na3VO4, leupeptin). The whole-cell lysates were immunoprecipitated with anti-Flag M2 agarose in the presence or absence of RNase A, ethidium bromide (EB). For mapping the regions within NSD2 responsible for its interaction with HSPA8 and RPL10, a Flag-tagged vector (Ctl) and a series of NSD2 isoforms were co-transfected with Myc-HSPA8 or Myc-RPL10 in HEK293T cells for co-immunoprecipitation experiments. The immunoprecipitated products were separated by SDS-PAGE and detected by immunoblotting with indicated antibodies.

### Immunoprecipitation-Mass Spectrometry

We used a combination of immunoprecipitations to study the NSD2 interaction partner, followed by qualitative mass spectrometry using LTQ-ESI-MS to measure the difference in the interaction between each isoform and contaminant protein. To identify proteins that specifically interact with each isoform of NSD2, we transfected cells expressing Isoform 1, Isoform 3, Isoform 5, or Isoform 7 expressing NSD2 into 293T cells, and 48 h later, lysed cells were collected. Protein and each experiment were repeated three times (biological replicate). After lysis, the total cell lysate was mixed for immunoprecipitation. These experiments were repeated, thus representing biological repeats. The immunoprecipitated protein was boiled and deformed and then subjected to SDS-PAGE to ensure the sample’s quality after immunoprecipitation. After determining the immunoprecipitation results of different transcripts, the silver strips were taken for mass spectrometry analysis. Each sample of mass spectrometry data was decontaminated and screened to obtain potential interacted proteins.

### Protein–Protein Interaction Data

The PPI data were downloaded from the BioGrid (v4.2.191) (Oughtred et al., 2019). BioGrid contains PPI with various detailed information, such as sources and experimental methods. We used the detailed PPI annotation provided by BioGrid to obtain direct physically interacted protein interactors. Network analyses and their visualizations were constructed by Cytoscape(Shannon et al., 2003).

### Gene Ontology Analysis

The Gene Ontology (GO) enrichment analysis of interested gene lists was performed by ClusterProfiler (Yu et al., 2012) R package. We tested if genes of interest enriched in any GO-BP pathway by hypergeometric test. Gene background was defined as all genes with GO annotation. *P*-value of hypergeometric tests was adjusted for multiple testing by the Benjamin–Hochberg method.

## Data Availability Statement

The original contributions presented in the study are included in the article/Supplementary Material, further inquiries can be directed to the corresponding authors.

## Author Contributions

GNL and LL conceived and directed the project. WW and WS curated and processed all of the data. JZ, YC, and LC participated in experiments. GNL, LL, WW, YC, and WS wrote and edited the manuscript. All authors read and approved the final manuscript.

## Conflict of Interest

The authors declare that the research was conducted in the absence of any commercial or financial relationships that could be construed as a potential conflict of interest.

## Footnotes

1 http://asia.ensembl.org
