# Supplementary material for: Alternatively Splicing Interactomes Identify Novel Isoform-Specific Partners for NSD2
Source: Front Cell Dev Biol. 2021 Feb 25;9:612019. doi: 10.3389/fcell.2021.612019 (PMC7947288; doi:10.3389/fcell.2021.612019)

Supplementary Figure 1. The flowchart and experiments of this study. (A) Alternative Splicing generates multiple protein isoforms within a precursor mRNA transcript, in which exons are differentially joined or skipped. (B) The interacted protein partners of protein isoforms are detected by using immunoprecipitation combined with mass spectrometry (IP-MS). (C) Isoform/exon specific protein partners are found based on the results of IP-MS, and their interactions are confirmed by Western Blotting.

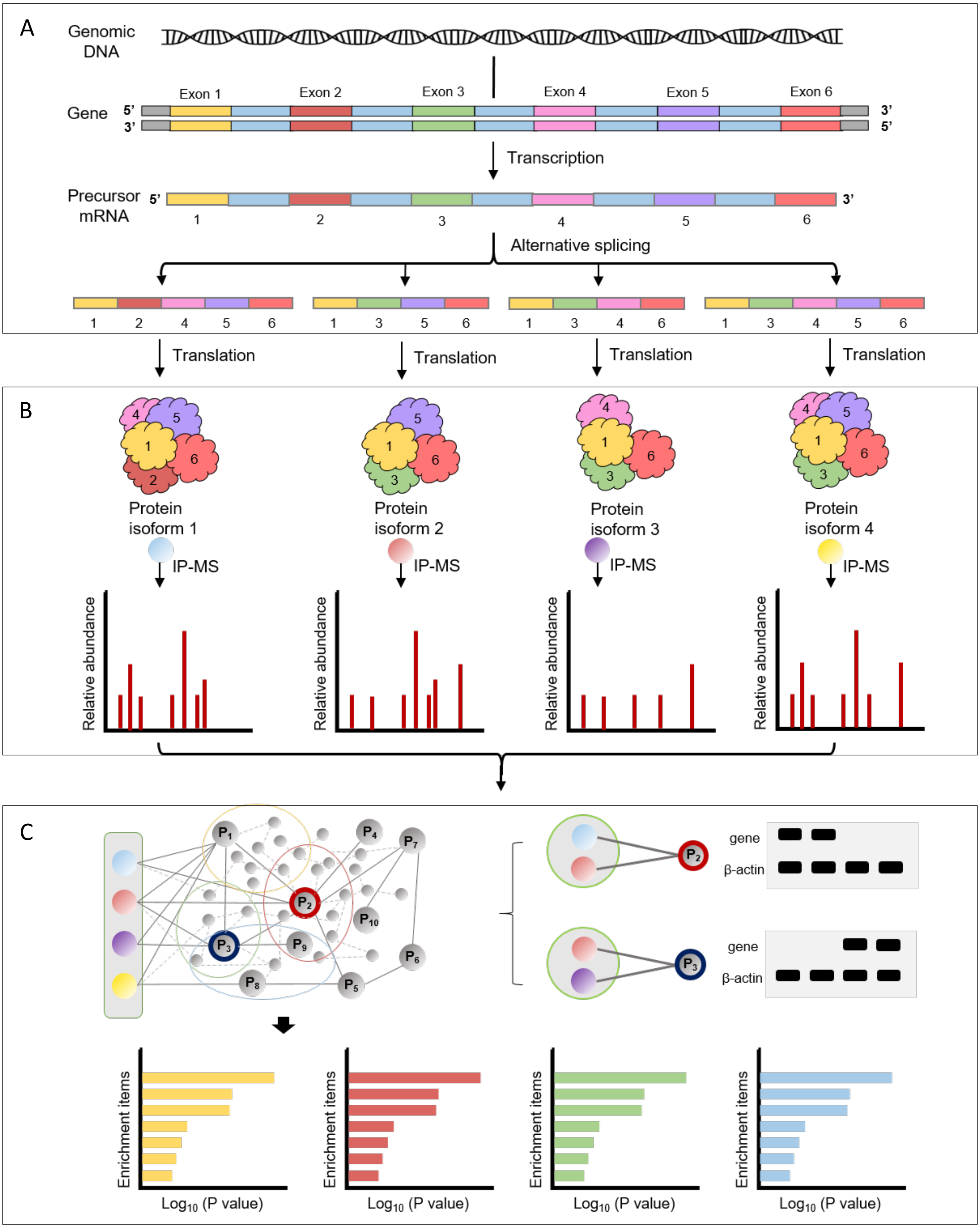

Supplementary Figure 2. The exon distributions of *NSD2* transcripts. Data Source: GTEx Analysis Release V8.

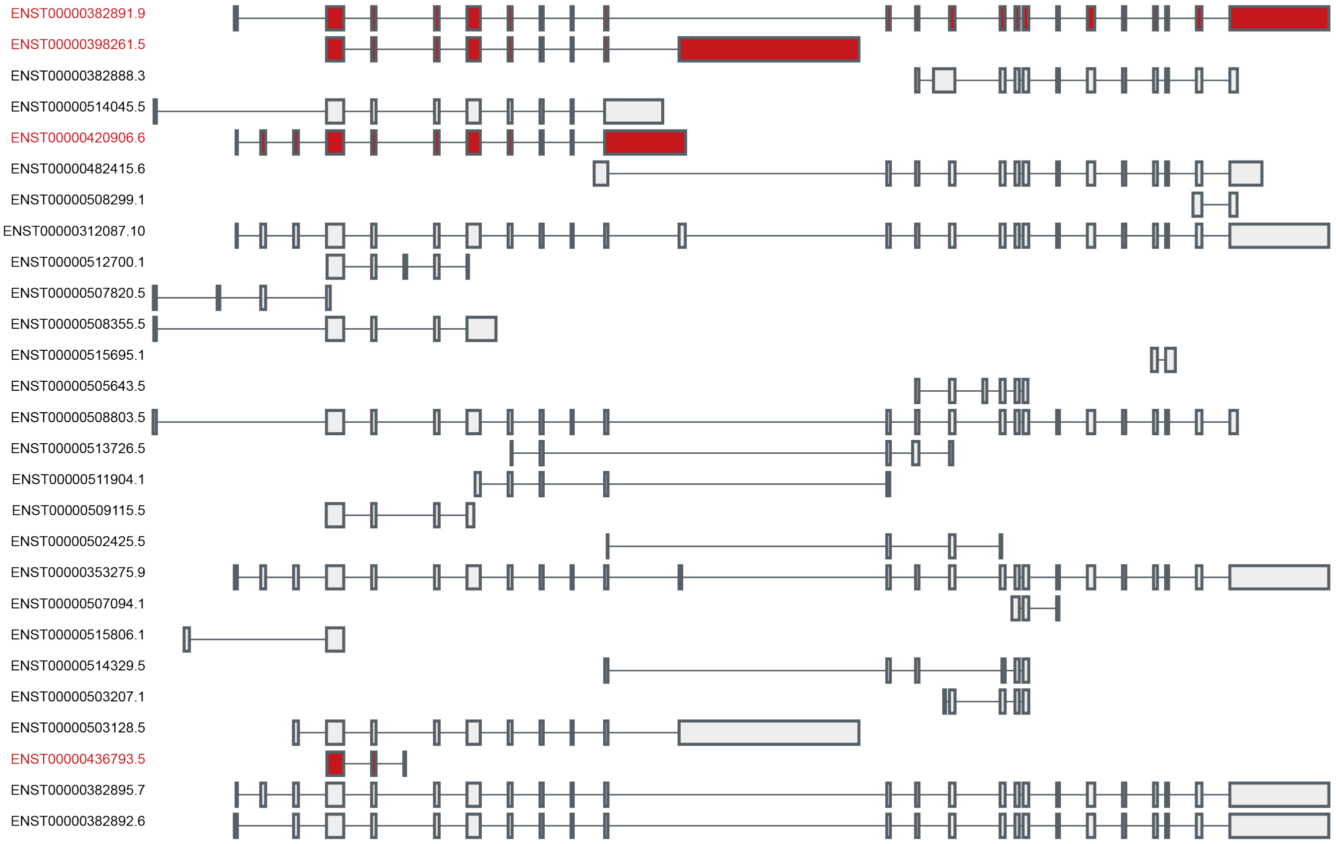

Supplementary Figure 3. Isoform 7 (ENST00000436793.5) of *NSD2* expressed in HEK293T, and the mRNA expression was normalized to Isoform 5 (ENST00000420906.6) .

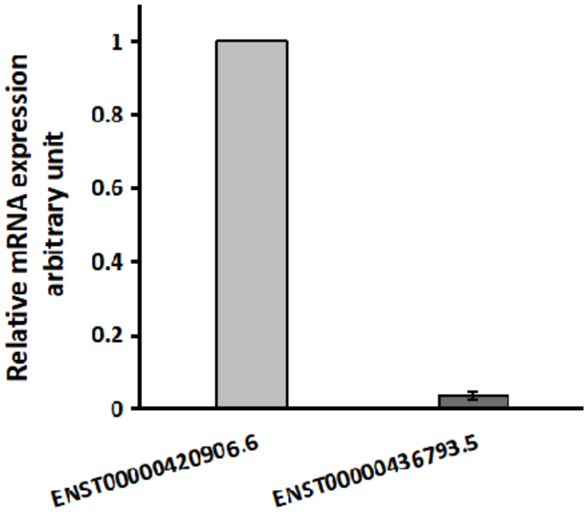

Supplement: Supplementary file 1 [file Data_Sheet_1.PDF]
